# Imeglimin may affect hemoglobin A1c accuracy via prolongation of erythrocyte lifespan in patients with type 2 diabetes mellitus: insights from the INFINITY clinical trial

**DOI:** 10.3389/fendo.2025.1699591

**Published:** 2025-10-24

**Authors:** Takeshi Osonoi, Shinichiro Shirabe, Miyoko Saito, Mitsuru Hosoya, Norie Watahiki, Nana Shiozawa, Satako Douguchi, Kensuke Ofuchi, Makoto Katoh

**Affiliations:** ^1^ Naka Kinen Clinic, Ibaraki, Japan; ^2^ Research Administration Center, Saitama Medical University, Saitama, Japan

**Keywords:** imeglimin, HbA1c, glycoalbumin, hemoglobin, erythrocyte lifespan, type 2 diabetes

## Abstract

**Background:**

Imeglimin is a novel type 2 diabetes (T2D) drug that is expected to improve mitochondrial function. In phase 3 trials, imeglimin demonstrated a gradual reduction in hemoglobin A1c (HbA1c), with a plateau at approximately 20–24 weeks. As erythrocyte lifespan may affect HbA1c accuracy, this clinical trial aimed to evaluate the potential discordance between HbA1c and other glycemic markers following imeglimin therapy and to explore whether imeglimin affects erythrocytes.

**Methods:**

This was a prospective, single-arm, open-label exploratory clinical trial (INFINITY study) conducted at Naka Kinen Clinic, Japan. Twenty-nine Japanese patients with inadequately controlled T2D received imeglimin 2,000 mg/day for six months. The primary endpoint was the change in hemoglobin concentration from baseline to Month 6. Secondary endpoints included serial changes in HbA1c, glycoalbumin (GA), 1,5-anhydroglucitol (1,5-AG), and erythrocyte lifespan, as well as comparison of glycemic reduction rates across these markers.

**Results:**

The change in hemoglobin concentration at 6 months was not statistically significant (mean ± SD: −0.2 ± 0.9 g/dL; p = 0.23). While HbA1c and GA decreased and 1,5-AG increased one month after imeglimin initiation, GA and 1,5-AG showed rapid changes compared to the gradual decrease in HbA1c. The divergence in reduction rates between HbA1c and the other markers persisted for up to two months. When erythrocyte lifespan was assessed using 3-month interval averages, significant prolongation was observed in both the 1–3 month and 4–6 month periods compared to the pre-treatment period (−2 to 0 months).

**Conclusion:**

This exploratory clinical trial suggests that imeglimin may prolong erythrocyte lifespan, resulting in disproportionately elevated HbA1c levels relative to true glycemic status. Reliance on HbA1c alone may underestimate imeglimin’s early glycemic effects, highlighting the value of alternative markers such as GA and 1,5-AG.

**Clinical trial registration:**

https://jrct.mhlw.go.jp/latest-detail/jRCTs031220489, Identifier jRCTs031220489.

## 1 Introduction

The number of patients with diabetes mellitus in Japan has increased with changes in lifestyle and the social environment. A recent survey estimated that there are 11 million people suspected of having diabetes, with a prevalence of 19.7% in men and 10.8% in women (1). In addition, diabetes mellitus affects 7.6% of adults aged 20–79 years (2), and improving diabetes care is an important issue.

Imeglimin is a drug for type 2 diabetes (T2D) treatment with a novel mechanism launched in Japan in September 2021. Although it is structurally related to metformin, unlike the biguanide class of metformin, imeglimin is a new class drug of tetrahydrotriazine-containing molecules called “glimins” (3). Imeglimin exerts its hypoglycemic effect by promoting glucose-stimulated insulin secretion and improving insulin resistance. Its mechanism of action may be mediated by an effect on the mitochondria (4), and it may improve mitochondrial function. It has also been suggested that imeglimin acts on the pancreas, skeletal muscle, and liver, three key organs involved in the pathophysiology of T2D, through targeting mitochondria and reducing oxidative stress (5).

In phase 3 clinical trials (TIMES 1–3) in Japanese patients with T2D, imeglimin, both as a single agent and in combination with other antidiabetic drugs, caused a slow decline in hemoglobin A1c (HbA1c) that plateaued around 24 weeks after administration (6–8). In a retrospective observational study, we reported a slower decrease in HbA1c compared to glycoalbumin (GA) and a divergence in the change rate between the two when imeglimin was administered to patients with T2D (9, 10). HbA1c levels reflect average blood glucose levels over the past 6–12 weeks, which is an indicator of long-term glycemic control, given that the erythrocyte lifespan is approximately 120 days. However, GA, a glycoprotein, is an indicator of short-term glycemic control because the half-life of albumin is shorter than that of erythrocytes at approximately 17–23 days (11). Therefore, GA accurately indicates changes in blood glucose levels within 2–3 weeks. However, when improvements in blood glucose levels occur in a short period of time, such as with antidiabetic drug treatment, changes in HbA1c are delayed, resulting in a transient divergence between the two. Comparing the rate of decrease from baseline in HbA1c and GA when dipeptidyl peptidase-4 (DPP-4) inhibitors are prescribed, a divergence is seen after 2 months, but thereafter, the two show almost identical trends (12). However, the divergence between HbA1c and GA observed with imeglimin treatment differed from the results of that report in both magnitude and duration, and short-term changes in blood glucose levels alone cannot explain this (9, 10, 12).

In general, factors that cause abnormal HbA1c values in the divergence between HbA1c and GA include shortening/lengthening of the erythrocyte lifespan (13) and increased/decreased erythrocyte production (14). One of the reasons for the higher HbA1c with imeglimin treatment compared to GA may be the prolonged erythrocyte lifespan. However, erythrocyte lifespan testing is not performed in routine clinical practice.

Prolonging the erythrocyte lifespan with imeglimin administration would result in a relatively high HbA1c and a slower rate of glycemic reduction than with GA or 1,5-anhydroglucitol (1,5-AG), suggesting that evaluating the glucose-lowering effect of imeglimin using HbA1c underestimates its effectiveness. There have been no reports, other than ours, on the divergence between HbA1c and GA or 1,5-AG on the glucose-lowering effect of imeglimin in patients with T2D under clinical conditions, or on its effect on erythrocytes (9, 10).

To address this issue, we conducted the INFINITY study, a prospective, single-arm, exploratory trial in patients with inadequately controlled T2D. The present report evaluates the temporal dynamics of HbA1c, GA, and 1,5-AG following imeglimin administration, and examines changes in erythrocyte lifespan and related hematologic indices. The aim of this study is to investigate whether imeglimin influences erythrocyte lifespan and to assess the implications for interpreting HbA1c levels in the early phase of treatment. To address this issue, we conducted the INFINITY study, a prospective, single-arm, open-label exploratory clinical trial in patients with inadequately controlled T2D.

The study protocol, including rationale and methods, has been previously published (15). The present article reports the clinical outcomes of that study.

## 2 Methods and analysis

### 2.1 Study design

This was a prospective, single-arm, open-label exploratory clinical trial conducted at Naka Kinen Clinic (Ibaraki, Japan).

### 2.2 Trial registration

The study protocol was registered in the Japan Registry of Clinical Trials (jRCTs031220489).

### 2.3 Ethics approval and consent

The trial was approved by the Certified Review Boards of Toho University (CRB3200009) and later Saitama Medical University (CRB3180022). All participants provided written informed consent before enrollment. The study was conducted in accordance with the Declaration of Helsinki and the Clinical Research Act of Japan.

### 2.4 Participants

Eligible participants were outpatients with T2D aged ≥20 years who were either treatment-naïve or receiving diet and exercise therapy alone, or in combination with metformin and/or α-glucosidase inhibitors (excluding acarbose). Additional inclusion criteria included an HbA1c between 6.5% and 8.5% and no changes in antidiabetic therapy for at least four weeks prior to consent. Key exclusion criteria included anemia (hemoglobin <13 g/dL for men and <12 g/dL for women), hypoalbuminemia (<3.0 g/dL), moderate to severe renal impairment (eGFR <45 mL/min/1.73 m²), hepatic dysfunction, and use of antiplatelet or anticoagulant medications.

### 2.5 Intervention

Participants received imeglimin (TWYMEEG^®^; 1,000 mg twice daily) for 6 months. A 2-month pre-observation period was used to establish baseline measurements, and a 3-month post-treatment follow-up period was included to assess potential reversibility of any treatment-related changes. During the observation and follow-up periods, no escalation or initiation of additional antidiabetic agents was allowed, except for continuation of stable doses of metformin or α-glucosidase inhibitors.

### 2.6 Endpoints and measurements

Primary endpoint: Change in hemoglobin concentration from baseline to Month 6.

Secondary endpoints: Changes in HbA1c, GA, 1,5-AG, erythrocyte lifespan (assessed by CO breath test), and hematologic indices.

### 2.7 Measurements

Erythrocyte lifespan, measured by exhaled carbon monoxide (CO) concentration using the Carbolizer system (Taiyo Co., Ltd., Japan) and calculated using the Strocchi formula (16). Fasting blood glucose (FBG), HbA1c, GA, and 1,5-AG levels, assessed at each time point and expressed as both absolute change and percentage reduction from baseline. Estimated HbA1c levels were calculated using data obtained from the flash glucose monitoring (FGM) system, FreeStyle Libre Pro (Abbott Diabetes Care Inc., Alameda, CA, USA), up to 2 months after imeglimin administration. Comparison of the reduction rates of HbA1c, GA, and 1,5-AG following imeglimin administration, with baseline values set to 100% (1,5-AG calculated as 100 minus the improvement rate). Additional hematologic indices: red and white blood cell counts, hematocrit, mean corpuscular volume (MCV), mean corpuscular hemoglobin (MCH), and MCH concentration (MCHC). Hemoglobin concentrations expressed as 3-month interval averages to assess temporal trends. No adjustment for multiplicity was performed for secondary outcomes, and p-values are considered descriptive.

### 2.8 Statistical analysis

The primary analysis was conducted in the full analysis set (FAS), defined as all patients who received at least one dose of imeglimin and had both baseline and post-baseline hemoglobin data. A per-protocol set (PPS) analysis was also performed as a sensitivity analysis. The primary endpoint was evaluated using a one-sample t-test comparing the mean change from baseline to Week 24 against the null hypothesis of zero change. Additionally, mixed-effects models for repeated measures (MMRM) were used to assess the longitudinal change in hemoglobin concentration, adjusting for baseline value and measurement time; this served as a sensitivity analysis. For secondary endpoints, descriptive statistics (mean ± SD or median [range]) were calculated for each time point. Glycemic reduction rates were calculated with baseline set as 100% and 1,5-AG expressed as “100 minus improvement rate.” Trends across markers were compared graphically. Erythrocyte lifespan and hemoglobin trends were assessed using paired t-tests comparing pre-treatment and post-treatment values (Months 1–3 and 4–6). No adjustments were made for multiple comparisons; all p-values are nominal, and analyses were performed using SAS version 9.4 (SAS Institute Inc.).

## 3 Results

### 3.1 Participant flow

**Figure 1:**
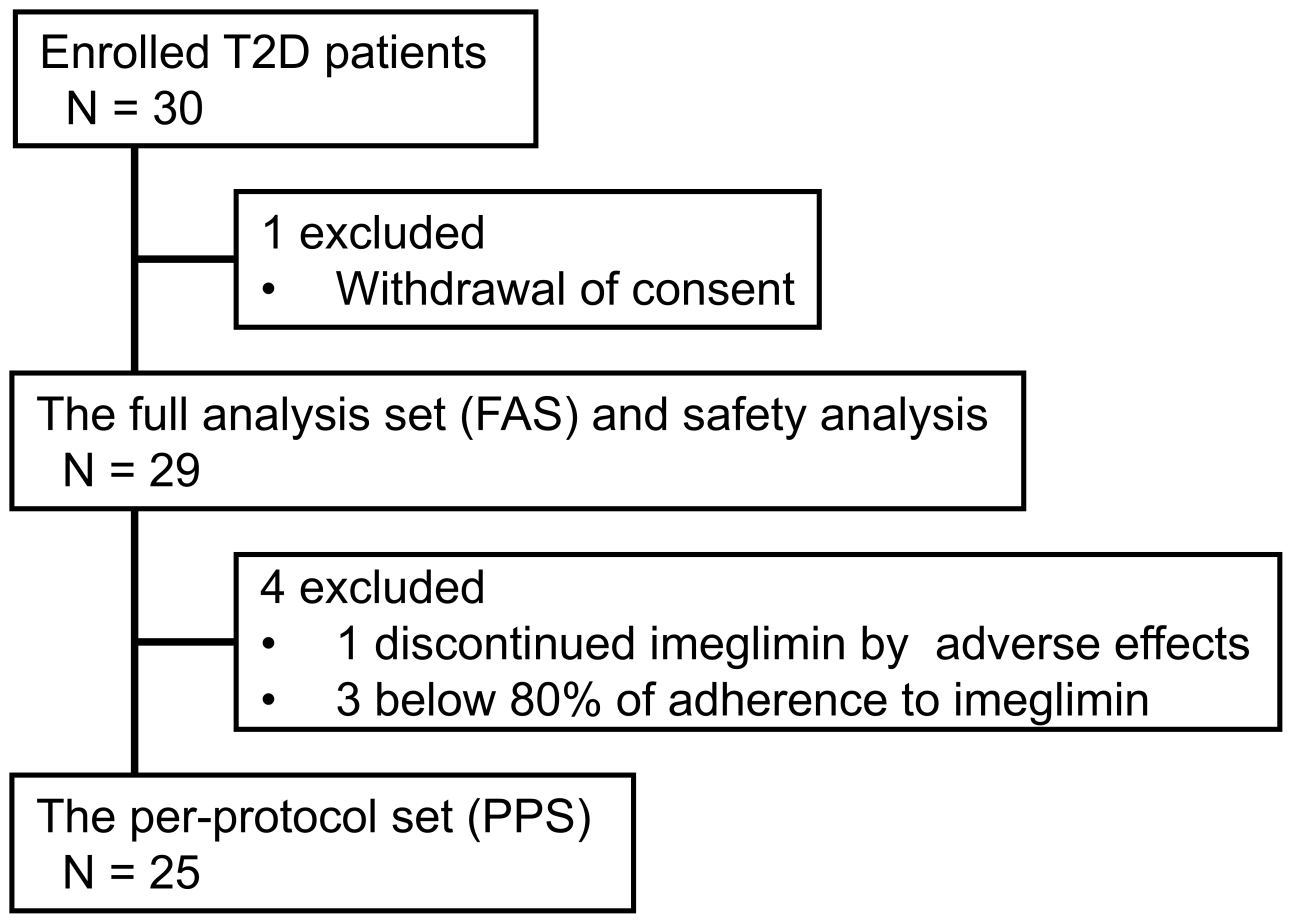
CONSORT-style flow diagram in the INFINITY study. T2D, type 2 diabetes.

A total of 30 patients were enrolled, achieving the target sample size. One patient withdrew consent before receiving imeglimin treatment. Twenty-nine patients were included in the FAS and safety analysis. One patient discontinued the study at Month 1 after initiating imeglimin due to gastrointestinal adverse effects. Three patients were excluded from the PPS because their adherence to the prescribed imeglimin dose and/or administration schedule was below 80%. As a result, the PPS included 25 patients. The participant flow is shown in **Figure 1** (CONSORT-style flow diagram adapted for single-arm trial).

### 3.2 Baseline characteristics

Baseline characteristics of the FAS (n = 29) are summarized in **Table 1**. There were 23 males (79.3%) and 6 females (20.7%), with a mean age of 64.7 ± 11.0 years, mean body mass index (BMI) of 25.3 ± 2.9 kg/m², mean FBG of 144.3 ± 24.6 mg/dL, mean HbA1c of 7.5 ± 0.6%, mean GA of 18.9 ± 2.9%, mean 1,5-AG of 9.1 ± 5.6 μg/mL, mean erythrocyte lifespan of 85.7 ± 20.3 days, mean hemoglobin of 14.7 ± 1.3 g/dL, mean erythrocyte count of 477.7 ± 52.6 ×10^4^/μL, mean white blood cell count of 55.0 ± 14.0 ×10²/μL, mean hematocrit of 43.7 ± 3.3%, mean corpuscular volume (MCV) of 91.8 ± 5.1 fL, mean corpuscular hemoglobin (MCH) of 30.8 ± 2.0 pg, and MCH concentration (MCHC) of 33.6 ± 1.0%.

**Table 1:** Baseline characteristics of all treated patients.

Variables	Full analysis set
n (male/female)	29 (23/6)
Age (years)	64.7 ± 11.0
BMI (kg/m^2^)	25.3 ± 2.9
FBG (mg/dL)	144.3 ± 24.6
HbA1c (%)	7.5 ± 0.6
GA (%)	18.9 ± 2.9
1, 5-AG (µg/mL)	9.1 ± 2.9
Erythrocyte lifespan (day)	85.7 ± 20.3
Hemoglobin (g/dL)	14.7 ± 1.3
Hematocrit (%)	43.7 ± 3.3
RBC count (10^4^/µL)	477.7 ± 52.6
WBC count (10^2^/µL)	55.0 ± 14.0
MCV (fL)	91.8 ± 5.1
MCH (pg)	30.8 ± 2.0
MCHC (%)	33.6 ± 1.0

Data are presented as mean ± standard deviation (SD).

BMI, body mass index; FBG, fasting blood glucose; HbA1c, hemoglobin A1c; GA, glycoalbumin; 1,5-AG, 1,5-anhydroglucitol; RBC, red blood cell; WBC, white blood cell; MCV, mean corpuscular volume; MCH, mean corpuscular hemoglobin; MCHC, mean corpuscular hemoglobin concentration.

The T2D patients in the study had hypertension (69.0%), dyslipidemia (86.2%), and cerebrovascular or cardiovascular disease (3.4%). The mean duration of T2D was 5.0 ± 4.8 years. At baseline, the following medications were prescribed for diabetes: α-glucosidase inhibitors alone (31.0%), metformin alone (24.1%) and its combination (3.4%), and treatment-naïve (41.4%); for hypertension: angiotensin receptor blocker (ARB) (41.4%), calcium channel blockers (CCB) (27.6%), diuretic (13.8%) and β-blocker (6.9%); for dyslipidemia: 3-hydroxy-3-methylglutaryl-coenzyme A reductase inhibitor (statin) (41.4%), ezetimibe (13.8%) and fibrate (3.4%).

### 3.3 Primary endpoint: hemoglobin concentration

The mean change in hemoglobin concentration from baseline to Month 6 was −0.2 ± 0.9 g/dL, which was not statistically significant (one-sample t-test: p = 0.23). A sensitivity analysis using a MMRM showed a least-squares mean difference of −0.2 g/dL (95% CI: −0.6 to 0.1, p = 0.17). Similar results were observed in the PPS (n = 25), indicating robustness of the findings.

### 3.4 Imeglimin-induced changes in glycemic markers: FBG, HbA1c, GA, and 1,5-AG

**Figure 2:**
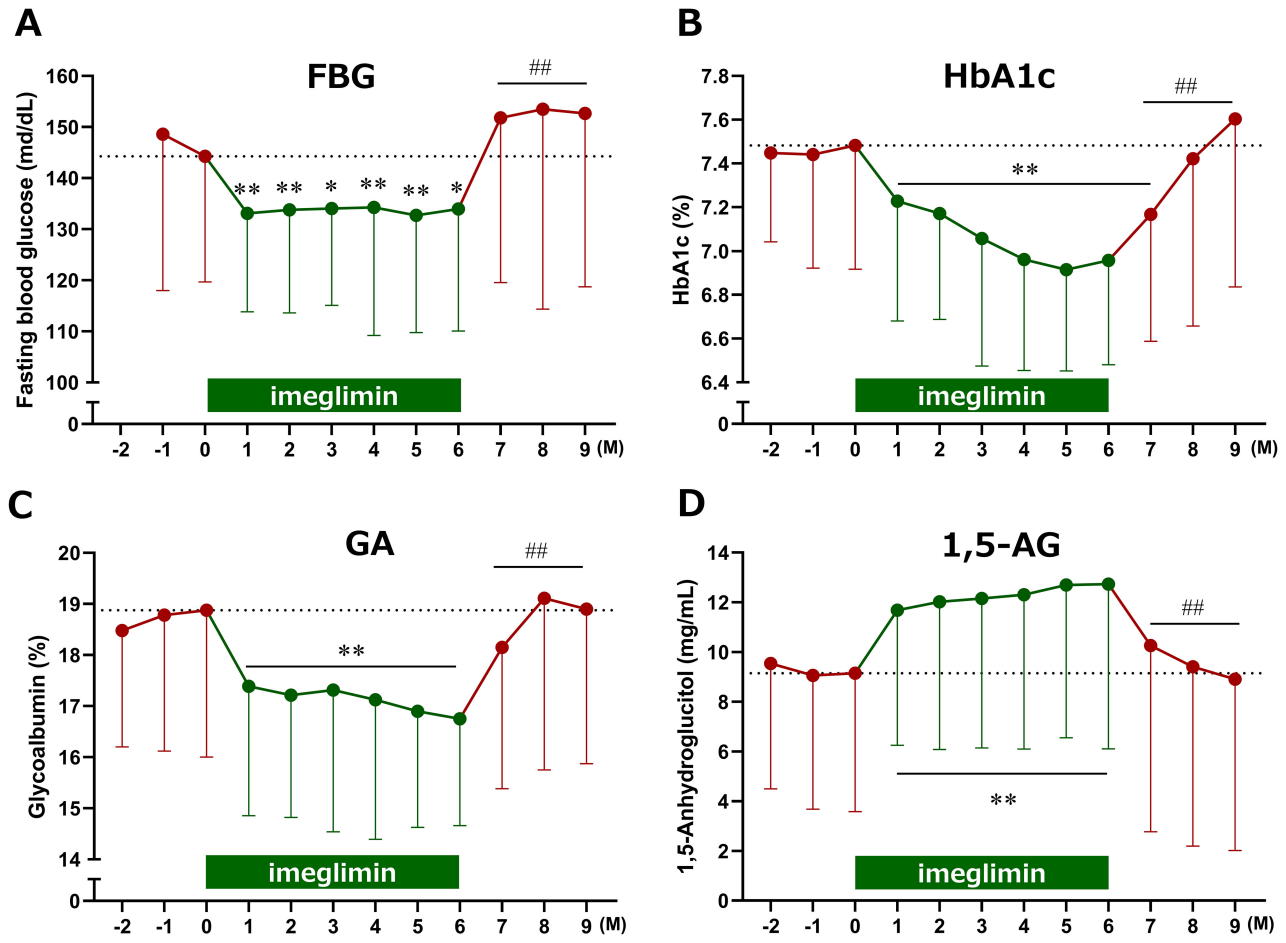
Time course of changes in glycemic markers following imeglimin initiation. **(A)** Fasting blood glucose (FBG), **(B)** HbA1c, **(C)** glycoalbumin (GA), and **(D)** 1,5-anhydroglucitol (1,5-AG). Data are expressed as mean ± SD. *p < 0.05, **p < 0.01 compared with baseline, ^##^p < 0.01 compared with the end of treatment (Month 6) (one-sample t-test). M, Month.

Following imeglimin administration, FBG, HbA1c, and GA significantly decreased, while 1,5-AG significantly increased as early as Month 1. These significant effects were sustained through Month 6 and rapidly returned to baseline after discontinuation of imeglimin (**Figure 2**). Notably, the patterns of glycemic improvement differed across markers: FBG, GA, and 1,5-AG demonstrated rapid changes within the first month, whereas HbA1c declined more gradually.

**Figure 3:**
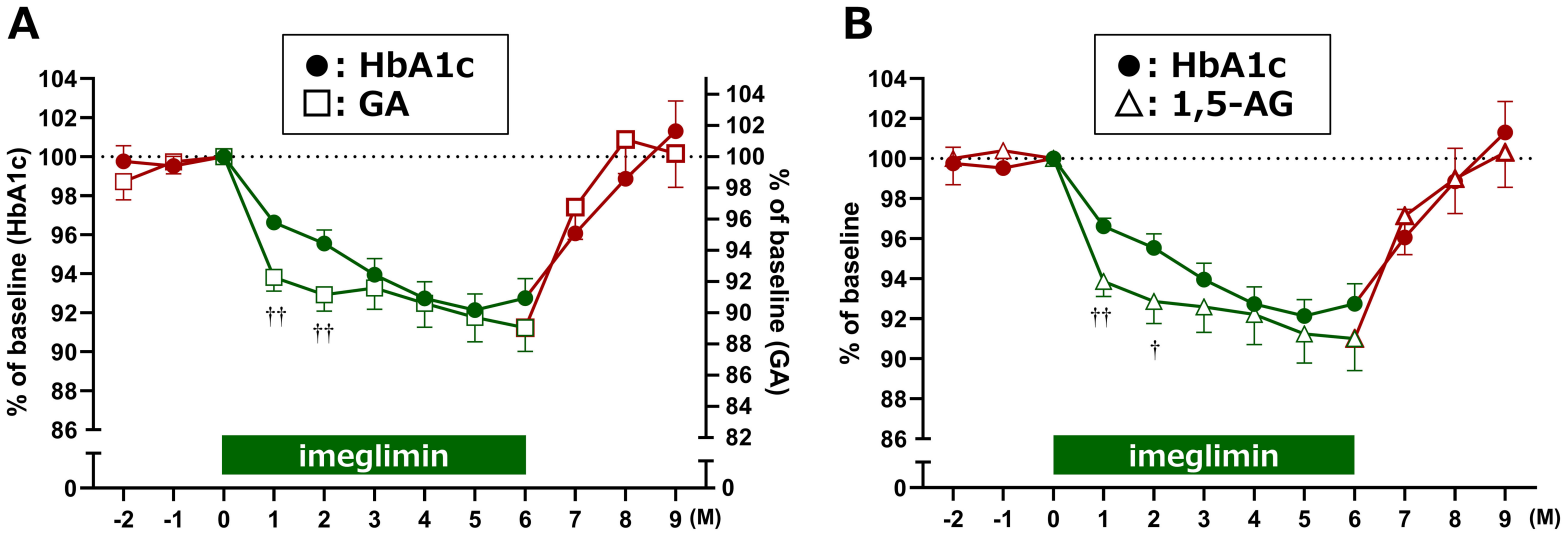
Relative reduction rates of glycemic markers after imeglimin initiation. Changes in **(A)** HbA1c vs. GA, and **(B)** HbA1c vs. 1,5-AG are shown as percentage reduction from baseline (set at 100%). Data are expressed as mean ± SD. ^†^p < 0.05, ^††^p < 0.01 for divergence between markers at each time point (one-sample t-test). M, Month.

To clarify these differences, glycemic reduction rates were calculated by setting baseline values to 100%. GA and 1,5-AG exhibited a sharp decrease at Month 1, whereas HbA1c showed a more gradual decline. The temporal changes in reduction rates between HbA1c and GA, as well as between HbA1c and 1,5-AG, demonstrated significant divergence at Months 1 and 2 after treatment initiation (**Figure 3**). Furthermore, estimated HbA1c values were calculated using data from the FGM system and compared with the measured HbA1c values. At baseline, estimated HbA1c (7.5 ± 0.9%, n = 28) and measured HbA1c (7.5 ± 0.6%, n = 29) were comparable. However, at 1 and 2 months after imeglimin administration, the estimated HbA1c values (6.6 ± 0.9%, n = 28; and 6.7 ± 0.9%, n = 27, respectively) were significantly lower than the measured HbA1c values (7.2 ± 0.5%, n = 29; and 7.2 ± 0.5%, n = 28, respectively) (p < 0.01 for both).

### 3.5 Effects of imeglimin on erythrocyte lifespan and erythrocyte-related parameters

**Figure 4:**
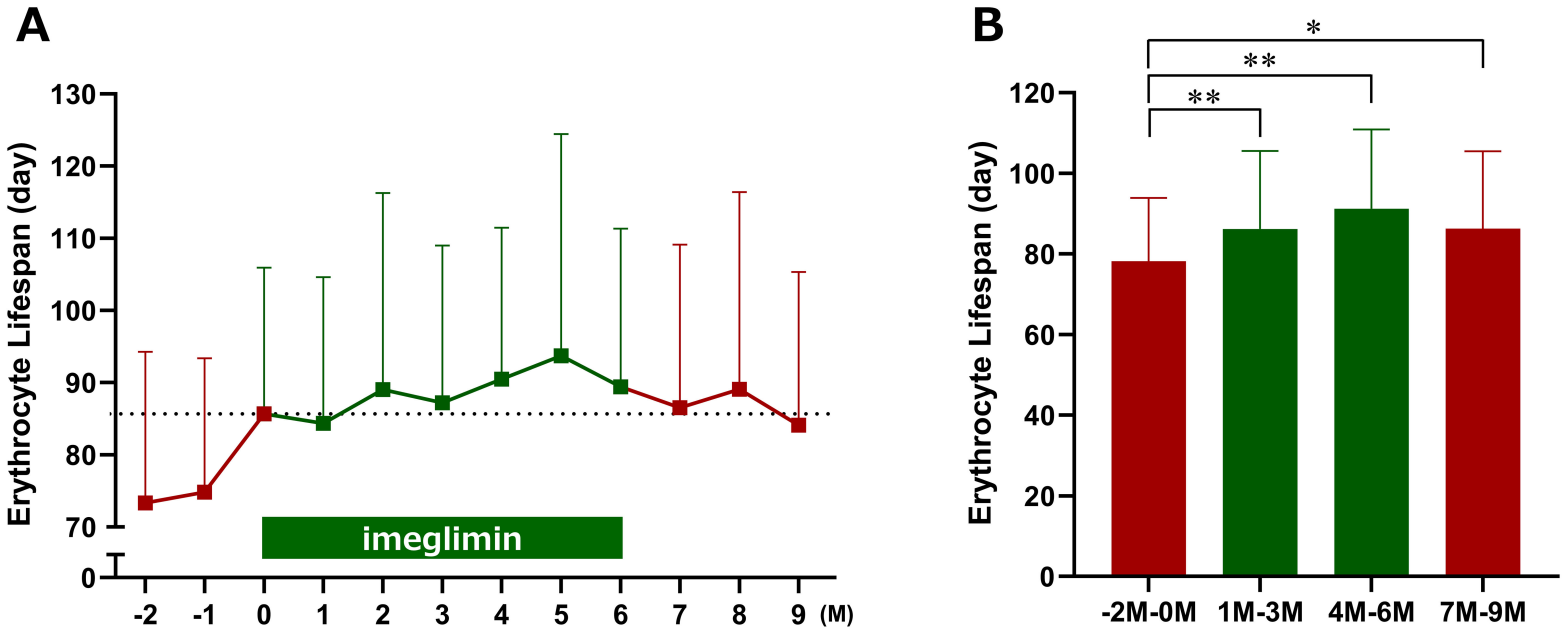
Effects of imeglimin on erythrocyte lifespan. **(A)** Changes in erythrocyte lifespan at each time point. **(B)** Mean erythrocyte lifespan over three-month intervals (−2 to 0 months, 1 to 3 months, 4 to 6 months, and 7 to 9 months). Data are expressed as mean ± SD. *p < 0.05, **p < 0.01 compared with the pre-treatment period (Months −2 to 0) (one-sample t-test). M, Month.

It is well recognized that HbA1c levels can appear elevated due to prolonged erythrocyte lifespan; therefore, we investigated the potential effects of imeglimin on erythrocytes. Although erythrocyte lifespan tended to be prolonged after imeglimin initiation compared with baseline, the change was not statistically significant (**Figure 4A**). To minimize the influence of seasonal variation and random fluctuations, we calculated averages over fixed 3-month intervals. Using this approach, erythrocyte lifespan was found to be significantly prolonged during both Months 1–3 and Months 4–6 of imeglimin treatment compared with the pre-treatment period (Months −2 to 0) (**Figure 4B**).

**Figure 5:**
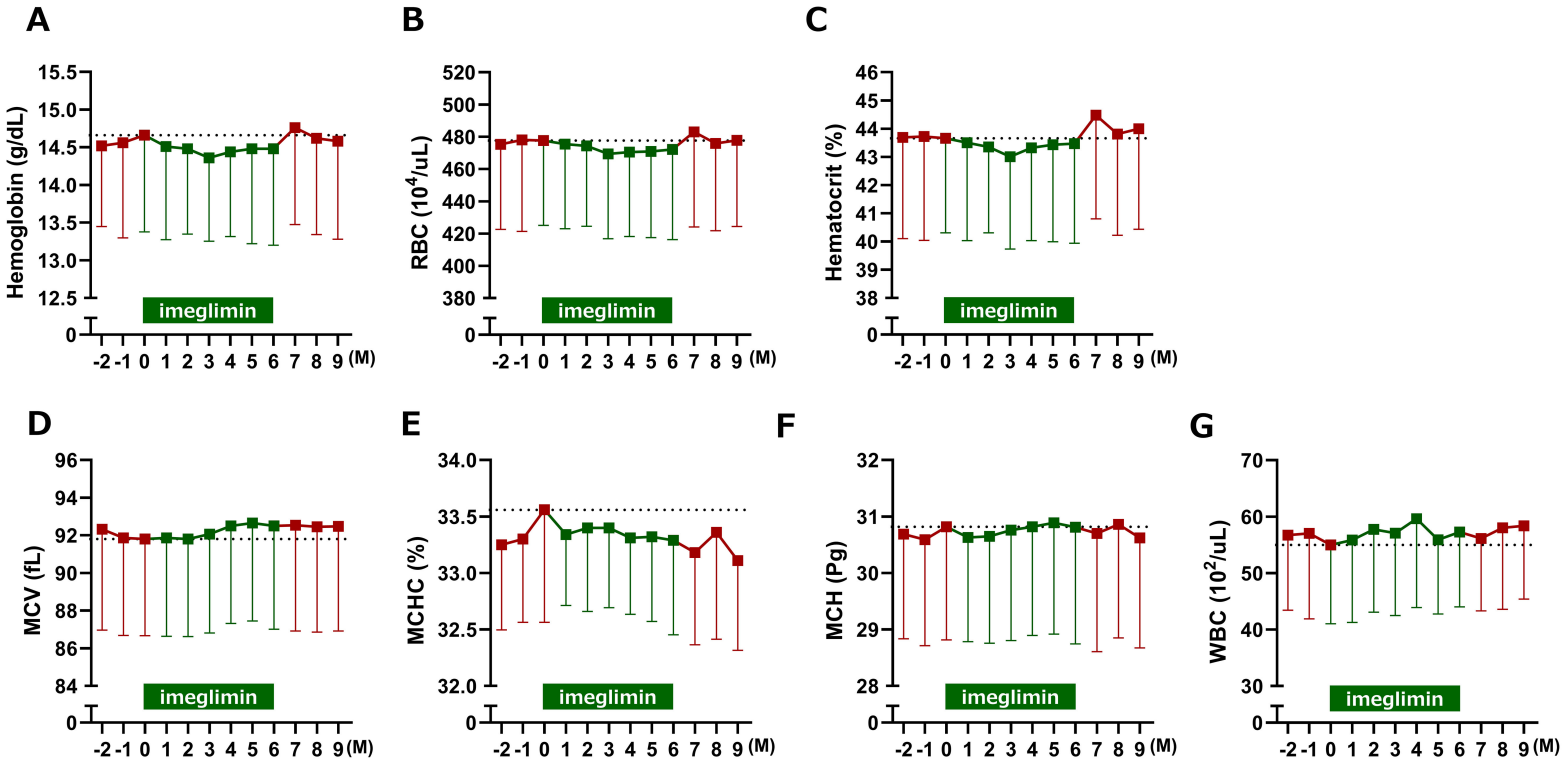
Changes in erythrocyte-related parameters after imeglimin initiation. **(A)** Hemoglobin, **(B)** red blood cell count (RBC), **(C)** hematocrit, **(D)** mean corpuscular volume (MCV), **(E)** mean corpuscular hemoglobin (MCH), **(F)** mean corpuscular hemoglobin concentration (MCHC), and **(G)** white blood cell count (WBC). Data are expressed as mean ± SD. No statistically significant changes were observed compared with baseline (one-sample t-test). M, Month.

**Figure 6:**
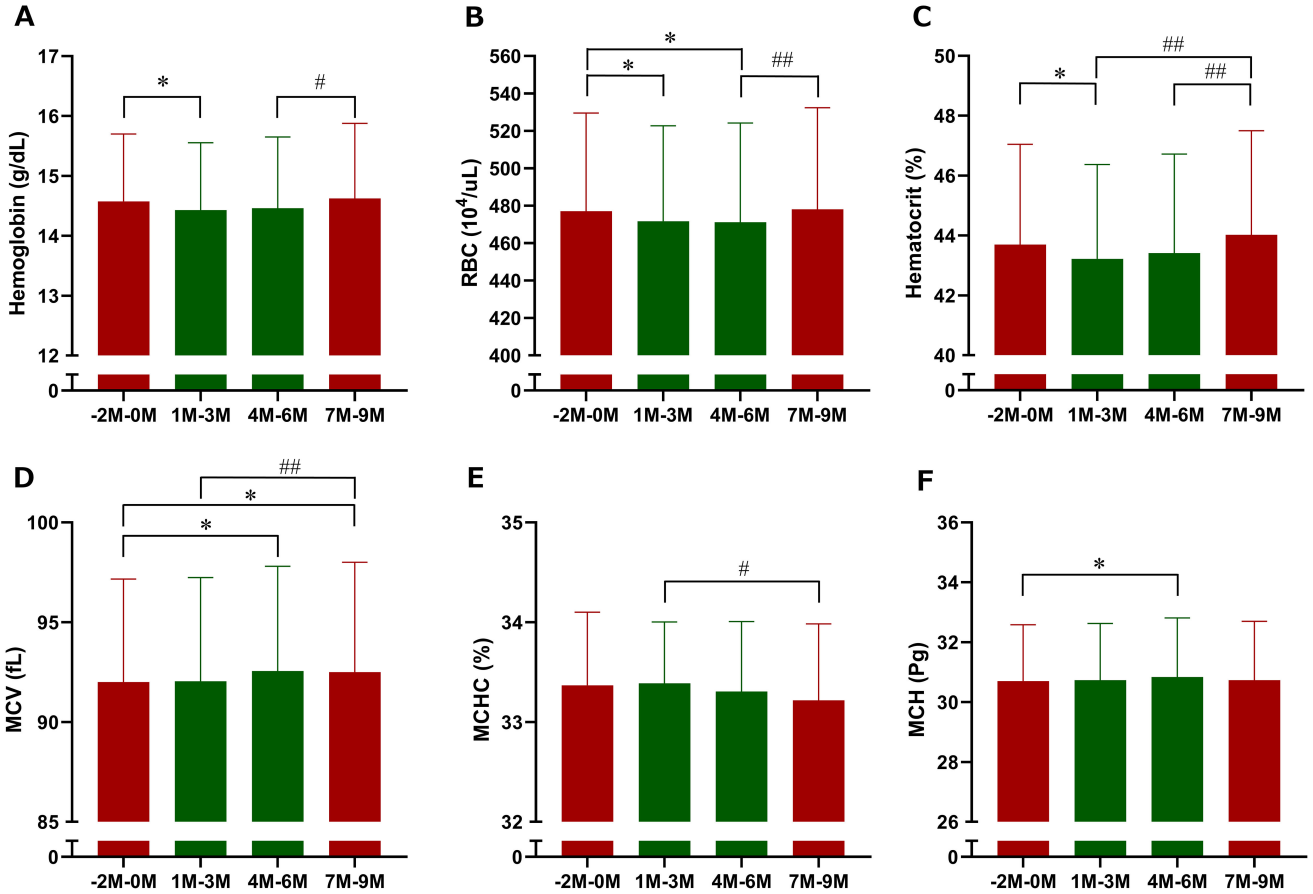
Three-month interval averages of erythrocyte-related parameters after imeglimin initiation. **(A)** Hemoglobin, **(B)** red blood cell count (RBC), **(C)** hematocrit, **(D)** mean corpuscular volume (MCV), **(E)** mean corpuscular hemoglobin (MCH), and **(F)** mean corpuscular hemoglobin concentration (MCHC). Data are expressed as mean ± SD. *p < 0.05 compared with the pre-treatment period (Months −2 to 0), ^#^p < 0.05, ^##^p < 0.01 compared with the follow-up period (Months 7 to 9) (one-sample t-test). M, Month.

Among erythrocyte-related parameters, hemoglobin, red blood cell count, hematocrit, and MCHC tended to decrease, whereas MCV tended to increase following imeglimin initiation compared with baseline; however, none of these changes reached statistical significance (**Figure 5**). When 3-month interval averages were applied, hemoglobin and hematocrit were significantly reduced during Months 1–3 of imeglimin treatment, red blood cell count was significantly reduced during both Months 1–3 and Months 4–6, and MCV and MCH were significantly reduced during Months 4–6 compared with the pre-treatment period; however, the overall impact on red blood cell indices was only marginal (**Figure 6**). Hemoglobin, red blood cell count, and hematocrit recovered after discontinuation of imeglimin. In addition, reticulocytes (% RBCs) were preliminarily assessed at baseline (1.87 ± 0.43, n=29), 1 month (1.86 ± 0.41, n=29), and 2 months (1.91 ± 0.43, n=28) after imeglimin administration, with no significant changes compared with baseline values.

As a negative control, white blood cell count was higher after initiation of imeglimin compared with baseline and remained higher than in the pre-treatment period when averaged over fixed 3 months; however, these differences were not statistically significant.

### 3.6 Safety and tolerability

In this study, AEs were observed in 20 patients (69.0%), and no patient experienced serious AEs. With respect to disease-related events, non-serious conditions occurred in 9 patients (31.0%), while no serious cases were reported. The majority of these events consisted of mild and transient gastrointestinal symptoms that developed within 2 months of initiating imeglimin treatment.

Body weight, BMI, and systolic blood pressure significantly decreased at Month 1 of imeglimin treatment; however, no clinically meaningful changes were observed thereafter. Diastolic blood pressure, heart rate, and lipid markers showed no clinically meaningful changes throughout the treatment period. Liver enzyme levels significantly decreased during imeglimin administration, with no evidence of worsening.

## 4 Discussion

The INFINITY study evaluated the comparative performance of clinical glycemic control markers during imeglimin treatment and its effects on erythrocytes in patients with type 2 diabetes mellitus. In this prospective exploratory study, the primary endpoint—the change in hemoglobin concentration at Month 6—did not reach statistical significance. However, when hemoglobin values were assessed using 3-month interval averages, a significant reduction was observed during Months 1–3 of imeglimin treatment compared with the pre-treatment period. Furthermore, imeglimin significantly prolonged erythrocyte lifespan during both Months 1–3 and Months 4–6. Although HbA1c decreased gradually through Month 6, the magnitude of reduction was smaller than that of GA and 1,5-AG, which improved rapidly after imeglimin initiation. These findings suggest that the relatively gradual change in HbA1c may be attributable, at least in part, to the prolongation of erythrocyte lifespan induced by imeglimin.

### 4.1 Effects on hemoglobin levels and erythrocyte lifespan

In this study, no significant change was observed when hemoglobin levels were assessed at Month 6 as a single time point; however, significant differences emerged when 3-month interval averages were applied, underscoring the importance of analyses that account for temporal variability. The hemoglobin results obtained in the present study are partially consistent with previous reports demonstrating significant reductions in 6-month average hemoglobin levels following imeglimin treatment (9, 10). Furthermore, the sample size calculation in this study was based on hemoglobin data averaged over 12 months (15). These findings suggest that imeglimin may exert only modest effects on hemoglobin levels, and that consistency with prior studies when temporal averaging is applied strengthens the reliability of our results.

In the present study, erythrocyte lifespan was indirectly assessed using a carbon monoxide (CO) breath test. The CO breath test is recognized as a valuable method for evaluating erythrocyte survival, characterized by its simplicity, rapidity, non-invasiveness, reproducibility, and high accuracy and sensitivity (17). The mean lifespan of erythrocytes is approximately 120 days, with a reported range of 70–140 days (17). In our study, the baseline erythrocyte lifespan was 85.7 ± 20.3 days. Previous reports have demonstrated that the mean erythrocyte lifespan in patients with type 2 diabetes (86.1 ± 18.1 days) is significantly shorter than that in healthy controls (103.6 ± 22.0 days) (18), which is consistent with our findings. It should be noted that the calculation of erythrocyte lifespan using the CO breath test incorporates hemoglobin values in the numerator of the formula (16), and thus tends to correlate with hemoglobin concentration. Nevertheless, in this study, erythrocyte lifespan was prolonged despite a reduction in hemoglobin levels, suggesting that imeglimin may contribute to lifespan extension through the suppression of erythrocyte degradation.

In this study, imeglimin administration was associated with a decrease in hemoglobin concentration and a concomitant prolongation of erythrocyte lifespan; however, the relationship between these two phenomena remains unclear. Previous reports have demonstrated that patients with high-altitude polycythemia (HAPC) or polycythemia vera (PV) exhibit significantly shorter erythrocyte lifespans compared with healthy individuals (17, 19), but the opposite scenario—prolongation of erythrocyte lifespan in the context of reduced hemoglobin concentration—has not been investigated in detail. Further studies are warranted to elucidate the potential relationship and underlying mechanisms linking hemoglobin reduction and erythrocyte lifespan extension.

### 4.2 Implications for HbA1c interpretation

In this study, the reduction in HbA1c was slower compared with GA and 1,5-AG, and this divergence persisted for up to 2 months after treatment initiation. Although this is the first prospective study to demonstrate a divergence among glycemic markers with imeglimin, the gradual decline in HbA1c has already been observed in the TIMES 1–3 studies (6–8) and is recognized as a well-established feature of imeglimin. Prolongation of erythrocyte lifespan provides a plausible mechanism to explain this divergence, as HbA1c reflects mean glycemia over the entire lifespan of circulating red blood cells; an extended lifespan allows for greater cumulative glycation, leading to higher HbA1c values relative to true glycemic status (13, 14). Consequently, reliance on HbA1c alone may underestimate the early glucose-lowering effect of imeglimin and could inadvertently lead to overtreatment, such as the addition of other antidiabetic agents, thereby increasing the risk of hypoglycemia. Therefore, GA and 1,5-AG should be used as complementary markers for the early evaluation of imeglimin’s therapeutic effects.

### 4.3 Potential mechanisms of erythrocyte effects

In the present study, the mechanisms underlying the imeglimin-induced prolongation of erythrocyte lifespan could not be elucidated. Imeglimin is a first-in-class oral antidiabetic agent that improves mitochondrial function through a novel mode of action. It has been reported that imeglimin promotes the conversion of nicotinamide (NAM) to nicotinamide mononucleotide (NMN) via increased expression of nicotinamide phosphoribosyltransferase (NAMPT) in the salvage pathway within pancreatic β-cells. Subsequently, NMN is converted to nicotinamide adenine dinucleotide (NAD^+^) by NMN adenylyltransferase (NMNAT), which may enhance both mitochondrial function and insulin secretion (4). Notably, NMNAT is also expressed in the cytoplasm of human erythrocytes (20). In mice lacking NMNAT, erythrocyte NAD^+^ levels are markedly reduced, accompanied by a shortened erythrocyte lifespan of approximately 10 days compared with about 60 days in wild-type mice, along with severe morphological abnormalities (21, 22).

In addition, erythrocytes rely entirely on glycolysis and the pentose phosphate pathway (PPP) for energy and redox balance. NAD^+^ availability is crucial for glycolytic flux through glyceraldehyde-3-phosphate dehydrogenase (GAPDH) (21) and for maintaining antioxidant capacity via NADPH generation in the PPP (23). An increase in intracellular NAD^+^ could therefore sustain glycolysis, enhance NADPH-dependent protection against oxidative stress, and preserve membrane deformability—factors that delay senescence and hemolysis (21). Moreover, imeglimin may enhance NAMPT and NMNAT activity in the NAD^+^ salvage pathway, stabilizing cytoskeletal and membrane proteins through NAD^+^-dependent deacetylation reactions (24). These observations collectively raise the possibility that imeglimin, by increasing erythrocyte NAD^+^ levels, may contribute to the prolongation of erythrocyte lifespan.

### 4.4 Clinical significance and future directions

HbA1c is the gold-standard biomarker for assessing long-term glycemic control and is widely used in both clinical trials and routine practice. However, its interpretation relies on the assumption of a stable erythrocyte lifespan. The present study demonstrated that particular caution is required when interpreting HbA1c levels during imeglimin treatment. Assessments based solely on HbA1c may underestimate therapeutic efficacy, whereas GA and 1,5-AG may more accurately capture early treatment effects. Moreover, the impact of imeglimin on erythrocyte lifespan may contribute to clinical benefits beyond glycemic control, representing an important topic for future investigation. Larger, long-term trials and mechanistic studies are warranted to confirm the present findings and to determine whether the modulation of erythrocyte lifespan constitutes a novel therapeutic aspect of imeglimin.

### 4.5 Limitations

This study has several limitations. First, it was conducted as a single-arm, single-center study with a small sample size, which limits the generalizability of the findings. Second, the analyses of 3-month interval averages and erythrocyte lifespan were exploratory and did not account for multiplicity; therefore, statistical significance should be interpreted with caution. Third, erythrocyte lifespan was estimated indirectly using exhaled carbon monoxide, which may be influenced by factors other than erythrocyte turnover. Fourth, this study did not include measurements of trace elements such as iron, copper, zinc, or RBC distribution width (RDW); thus, their potential influence on the observed hematologic changes cannot be entirely excluded. Nevertheless, participants exhibited no clinical signs of iron deficiency or anemia, and mean hemoglobin levels remained within the normal range. Taken together, as an exploratory clinical trial, the results should be considered preliminary and require validation in larger, controlled studies.

## 5 Conclusion

This exploratory clinical trial suggests that imeglimin may prolong erythrocyte lifespan, which could result in disproportionately elevated HbA1c levels relative to actual glycemic status. Such a discrepancy may lead to underestimation of imeglimin’s early glycemic effects when relying solely on HbA1c as a marker of treatment efficacy. Our findings highlight the importance of incorporating alternative glycemic markers, such as GA and 1,5-AG, particularly during the early phase of imeglimin treatment or in patients with potential alterations in erythrocyte kinetics. Further mechanistic and longitudinal studies are warranted to confirm these observations and to optimize glycemic monitoring strategies for patients receiving imeglimin.

## Data Availability

The data supporting the findings of this study are available from the corresponding author upon reasonable request. Due to ethical restrictions and participant confidentiality, access to the data may be limited and will be provided in accordance with institutional guidelines and applicable data-sharing policies. Requests to access the datasets should be directed to m_katou@saitama-med.ac.jp.
