# Supplementary material for: Imeglimin may affect hemoglobin A1c accuracy via prolongation of erythrocyte lifespan in patients with type 2 diabetes mellitus: insights from the INFINITY clinical trial
Source: Front Endocrinol (Lausanne). 2025 Oct 24;16:1699591. doi: 10.3389/fendo.2025.1699591 (PMC12591969; doi:10.3389/fendo.2025.1699591)
Supplement: Supplementary file 1 [file Table1.docx]

# CONSORT 2010 Checklist for the INFINITY Clinical Trial

(Single-arm, open-label, prospective exploratory study; non-randomized)

| Section/Topic | Item No | Checklist item | Reported on page/section |
| --- | --- | --- | --- |
| Title and abstract | 1a | Identification as a randomized trial in the title | Not applicable (single-arm study) |
|  | 1b | Structured summary of trial design, methods, results, and conclusions (see CONSORT for abstracts) | Abstract |
| Introduction | 2a | Scientific background and explanation of rationale | Introduction |
|  | 2b | Specific objectives or hypotheses | Introduction |
| Methods: Trial design | 3a | Description of trial design (such as parallel, factorial) including allocation ratio | Methods – Study design |
|  | 3b | Important changes to methods after trial commencement, with reasons | Not applicable / none |
| Participants | 4a | Eligibility criteria for participants | Methods – Participants |
|  | 4b | Settings and locations where the data were collected | Methods – Study design |
| Interventions | 5 | The interventions for each group with sufficient details to allow replication | Methods – Intervention |
| Outcomes | 6a | Completely defined pre-specified primary and secondary outcome measures, including how and when they were assessed | Methods – Endpoints |
|  | 6b | Any changes to trial outcomes after the trial commenced, with reasons | Not applicable / none |
| Sample size | 7a | How sample size was determined | Methods – Sample size description |
|  | 7b | When applicable, explanation of any interim analyses and stopping guidelines | Not applicable |
| Randomisation | 8a | Method used to generate the random allocation sequence | Not applicable |
|  | 8b | Type of randomisation; details of any restriction (such as blocking and block size) | Not applicable |
| Allocation concealment mechanism | 9 | Mechanism used to implement the random allocation sequence | Not applicable |
| Implementation | 10 | Who generated the allocation sequence, who enrolled participants, and who assigned participants to interventions | Not applicable |
| Blinding (masking) | 11a | If done, who was blinded after assignment to interventions (e.g., participants, care providers, outcome assessors) | Not applicable |
|  | 11b | If relevant, description of the similarity of interventions | Not applicable |
| Statistical methods | 12a | Statistical methods used to compare groups for primary and secondary outcomes | Methods – Statistical analysis |
|  | 12b | Methods for additional analyses, such as subgroup analyses and adjusted analyses | Methods – Statistical analysis |
| Results: Participant flow | 13a | For each group, numbers of participants who were randomly assigned, received intended treatment, and were analysed | Results – Participant flow |
|  | 13b | For each group, losses and exclusions after randomisation, together with reasons | Results – Participant flow |
| Recruitment | 14a | Dates defining the periods of recruitment and follow-up | Trial status |
|  | 14b | Why the trial ended or was stopped | Trial status (completed as planned) |
| Baseline data | 15 | A table showing baseline demographic and clinical characteristics for each group | Results – Baseline characteristics |
| Numbers analysed | 16 | For each group, number of participants included in each analysis and whether the analysis was by original assigned groups | Results – Participant flow, FAS and PPS |
| Outcomes and estimation | 17a | For each primary and secondary outcome, results for each group, and the estimated effect size and its precision (e.g., 95% CI) | Results – Primary endpoint, Secondary endpoints |
|  | 17b | For binary outcomes, presentation of both absolute and relative effect sizes | Not applicable |
| Ancillary analyses | 18 | Results of any other analyses performed, including subgroup analyses and adjusted analyses, distinguishing pre-specified from exploratory | Not performed / Not applicable |
| Harms | 19 | All important harms or unintended effects in each group | Results – Safety and tolerability |
| Discussion | 20 | Trial limitations, addressing sources of potential bias, imprecision, and, if relevant, multiplicity of analyses | Discussion – Limitations |
|  | 21 | Generalisability (external validity, applicability) of the trial findings | Discussion |
|  | 22 | Interpretation consistent with results, balancing benefits and harms, and considering other relevant evidence | Discussion |
| Other information | 23 | Registration number and name of trial registry | Title page, Methods – Trial registration |
|  | 24 | Where the full trial protocol can be accessed, if available | Methods – Reference to published protocol |
|  | 25 | Sources of funding and other support; role of funders | Funding section |
